# Balanced double aortic arch with late-onset symptoms detected on ^18^F-FDG PET/CT in an octogenarian with advanced prostate cancer: A case report

**DOI:** 10.1097/MD.0000000000051051

**Published:** 2026-10-09

**Authors:** Qiang Wei, Panhui Liu, Yinghua Wu

**Affiliations:** a School of Medicine and Life Sciences, Chengdu University of Traditional Chinese Medicine, Chengdu, Sichuan, China; b Hospital of Chengdu University of Traditional Chinese Medicine, Chengdu, Sichuan, China.

**Keywords:** ^18^F-FDG PET/CT, double aortic arch, octogenarian, swallowing discomfort, vascular ring

## Abstract

**Rationale:**

Double aortic arch (DAA) is a rare congenital vascular anomaly usually diagnosed in infancy. Adult or elderly presentation is uncommon, and balanced-type DAA is relatively rare. On oncologic ^18^F-fluorodeoxyglucose (FDG) positron emission tomography (PET)/computed tomography (CT), a mediastinal tubular structure with blood-pool-level FDG uptake may provide an important clue to a vascular anomaly.

**Patient concerns:**

An 80-year-old man with biopsy-proven advanced prostate cancer underwent ^18^F-FDG PET/CT for systemic staging. He had dysuria and back pain for more than 1 year, with mild intermittent swallowing discomfort.

**Diagnoses:**

PET/CT showed multiple FDG-avid bone metastases. Axial and coronal PET/CT images demonstrated bilateral aortic arch components adjacent to the tracheoesophageal region, with FDG uptake equivalent to the aortic blood pool. Three-dimensional volume rendering confirmed balanced-type DAA forming a complete vascular ring.

**Interventions:**

No DAA-directed intervention was performed. Additional CT angiography was not pursued because the PET/CT findings were characteristic and further vascular imaging was unlikely to alter management.

**Outcomes:**

Conservative management was recommended. At approximately 6 months after PET/CT, the patient’s intermittent swallowing discomfort remained stable, and no DAA-directed intervention was performed.

**Lessons:**

Balanced-type DAA may be incidentally detected on oncologic ^18^F-FDG PET/CT. Recognition of a tubular mediastinal structure continuous with the aortic system and showing blood-pool-level FDG uptake may help avoid misinterpretation as malignant mediastinal disease.

## 1. Introduction

Double aortic arch (DAA) is a rare congenital vascular anomaly and an important cause of a complete vascular ring.^[1,2]^ It results from persistence of both fourth primitive aortic arches and the related dorsal aortic segments, creating a vascular ring that may compress the trachea and esophagus.^[2,3]^

Most affected patients are diagnosed in infancy because of respiratory or feeding symptoms caused by tracheoesophageal compression.^[4]^ Adult-onset or elderly presentation is unusual, and balanced-type DAA with similar diameters of both arches is relatively uncommon.^[5–8]^ We report an octogenarian with advanced prostate cancer in whom balanced-type DAA was detected on ^18^F-fluorodeoxyglucose (FDG) positron emission tomography (PET)/computed tomography (CT) performed for oncologic staging.

## 2. Case presentation

An 80-year-old man with biopsy-proven prostate cancer underwent ^18^F-FDG PET/CT for systemic staging. His primary symptoms were dysuria and back pain for more than 1 year, accompanied by self-reported mild, intermittent swallowing discomfort. Maximum-intensity projection PET imaging showed extensive FDG-avid osseous disease, consistent with advanced metastatic prostate cancer and providing the oncologic context for the examination (Fig. 1A).

**Figure 1.**
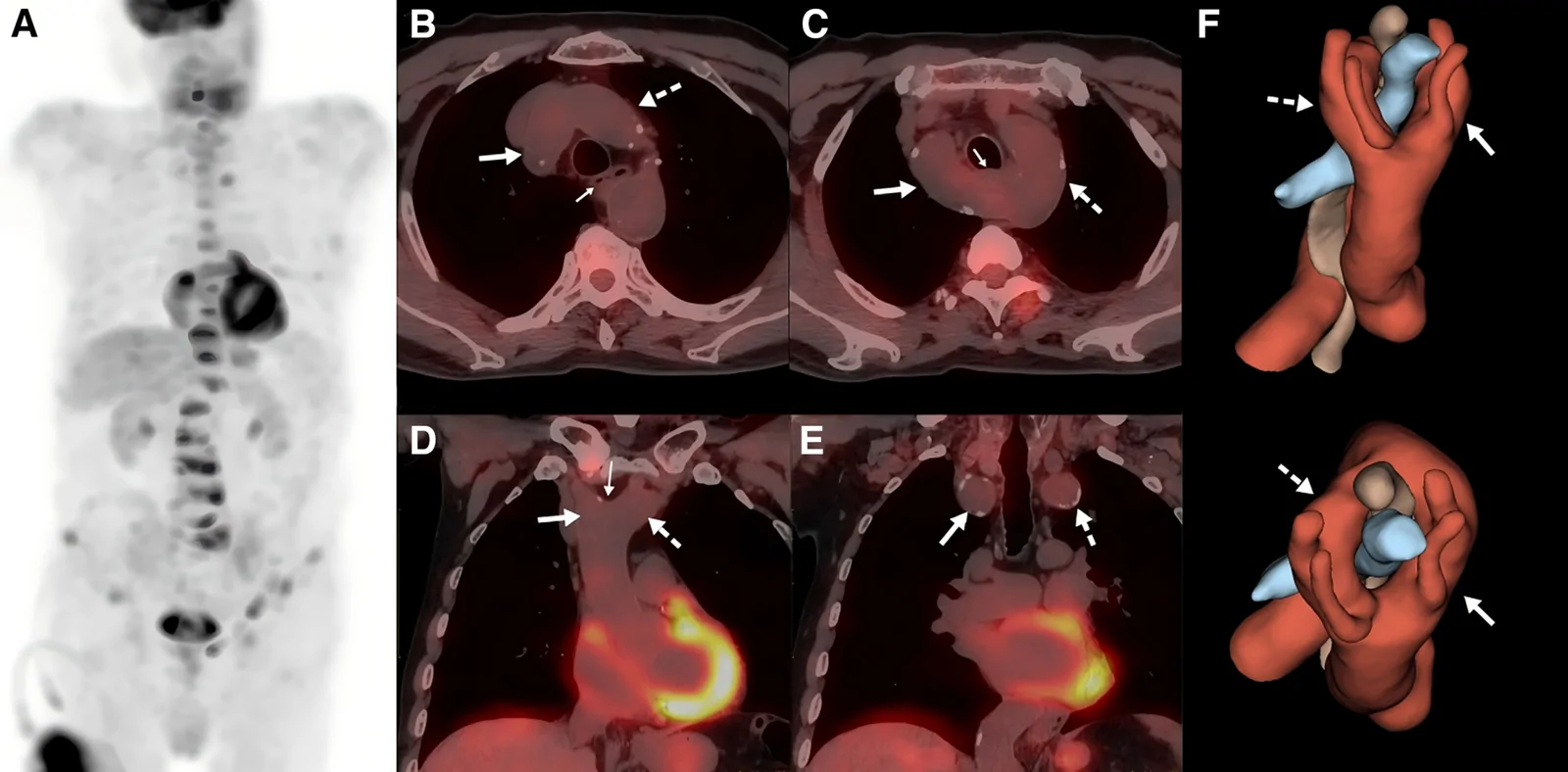
^18^F-FDG PET/CT findings of balanced-type double aortic arch. Throughout panels (B–F), solid arrows indicate the right aortic arch, whereas dashed arrows indicate the left aortic arch. (A) Maximum-intensity-projection PET image showing multiple FDG-avid bone metastases; (B and C) axial PET/CT fusion images demonstrating the bilateral aortic arches surrounding the esophagus (thin arrows), with FDG uptake comparable to the aortic blood pool; (D and E) Coronal PET/CT fusion images showing the course and continuity of the bilateral aortic arches with the aortic system, the thin arrow in panel (D) indicates the bifurcation of the ascending aorta into the right and left aortic arches; (F) 3D volume-rendered reconstructions showing the bilateral aortic arches forming a complete vascular ring. In panel (F), the aortic system, segmented trachea, and segmented esophagus are rendered in red, light blue, and gray, respectively. 3D = three-dimensional, CT = computed tomography, DAA = double aortic arch, FDG = fluorodeoxyglucose, PET = positron emission tomography.

During image interpretation, an incidental upper mediastinal vascular structure was noted. Axial PET/CT fusion images demonstrated bilateral aortic arch components adjacent to the tracheoesophageal region, without focal hypermetabolic uptake beyond the blood-pool level (Fig. 1B and 1C). Coronal PET/CT fusion images further showed continuity of the bilateral arch components with the aortic system (Fig. 1D and 1E). On the low-dose CT component, the structure showed an attenuation of approximately 30 HU, and PET images showed low-grade FDG uptake in the bilateral aortic arch components (maximum standardized uptake value, 1.3), comparable to that of the descending thoracic aortic blood pool (maximum standardized uptake value, 1.38). Segmentation-based three-dimensional (3D) volume-rendered reconstruction generated using 3D Slicer software (version 5.10.1) demonstrated bilateral aortic arches forming a complete vascular ring, with comparable calibers of both arches and a symmetric 4-vessel configuration, consistent with balanced-type DAA (Fig. 1F).

Because the swallowing discomfort was mild and intermittent, the patient had advanced malignancy with extensive metastatic disease, and the PET/CT morphology and blood-pool FDG uptake pattern were sufficient to support the diagnosis, additional computed tomography angiography was deferred because it was unlikely to alter clinical management. Conservative management with symptom-based clinical follow-up was recommended. At telephone follow-up approximately 6 months after the PET/CT examination, the patient reported that his intermittent swallowing discomfort remained stable. He continued to receive endocrine therapy for advanced prostate cancer and bone-targeted therapy for osseous metastases; no DAA-directed intervention had been performed.

## 3. Discussion

DAA is a rare congenital vascular anomaly and is considered the most common symptomatic vascular ring.^[1,2]^ Morphologically, many cases have a dominant arch, whereas a balanced configuration with comparable diameters of both arches is less common.^[5,8]^ In the present case, multiplanar PET/CT review and segmentation-based 3D volume rendering demonstrated a complete vascular ring and a symmetric 4-vessel configuration, allowing confident recognition of balanced-type DAA.

The clinical presentation of DAA depends on the degree of compression of adjacent structures. Dysphagia can result from a broad range of intrinsic and extrinsic causes, including vascular compression.^[9]^ In patients with advanced malignancy, nonspecific swallowing symptoms and mediastinal abnormalities may be clinically attributed to malignant disease or lymphadenopathy. However, mediastinal and hilar lymph node metastases from prostate adenocarcinoma are rare and may create diagnostic uncertainty.^[10]^ In this case, the tubular morphology, continuity with the aortic system, and FDG uptake equivalent to aortic blood pool supported a vascular rather than malignant etiology.

The main differential diagnoses of an upper mediastinal tubular structure on PET/CT include vascular variants, dilated mediastinal veins, and lymphadenopathy. A vascular origin is suggested by a smooth tubular configuration, continuity with the great vessels on multiplanar images, and FDG uptake comparable to the blood pool. Dilated mediastinal veins generally follow the expected venous anatomy, whereas lymphadenopathy usually appears as discrete or confluent soft-tissue nodules without continuity with the vascular lumen.

Most DAA patients are diagnosed during infancy, whereas adult-onset symptoms and incidental detection in elderly patients are uncommon.^[4,6,7]^ In this octogenarian, late-onset swallowing discomfort may have been related to age-associated vascular degeneration and atherosclerosis, which could increase the mechanical rigidity of the vascular ring.^[11]^ In addition, tumor-related catabolic changes and possible loss of mediastinal fat buffering may have contributed to delayed clinical presentation.^[12]^ These mechanisms may provide a plausible explanation for the late-onset symptoms, although further vascular assessment was not performed.

Computed tomography angiography or magnetic resonance angiography should be considered when the vascular anatomy remains uncertain, when dysphagia or respiratory symptoms are progressive, when clinically significant tracheoesophageal compression is suspected, or when surgical intervention is being considered.

This case emphasizes the value of integrating anatomic and metabolic information on ^18^F-FDG PET/CT. When a mediastinal abnormality demonstrates characteristic vascular morphology and blood-pool-level FDG uptake, a congenital vascular anomaly should be considered even in an elderly patient with advanced cancer. Recognition of this pattern may prevent misattribution of a vascular ring to malignant mediastinal disease and may guide conservative management when symptoms are mild and additional imaging would not change treatment.

## Acknowledgments

This work was supported by the Joint Innovation Fund of Health Commission of Chengdu and Chengdu University of Traditional Chinese Medicine (Grant No. WXLH202403177).

## Author contributions

**Data curation:** Qiang Wei, Panhui Liu.

**Investigation:** Qiang Wei, Panhui Liu.

**Supervision:** Yinghua Wu.

**Validation:** Qiang Wei.

**Visualization:** Panhui Liu.

**Writing – original draft:** Qiang Wei.

**Writing – review & editing:** Yinghua Wu.
